# Proton beam therapy for a patient with prostatic rhabdomyosarcoma: a case report and review of the literature

**DOI:** 10.3389/fonc.2025.1490098

**Published:** 2025-06-10

**Authors:** Wencui Yang, Junetsu Mizoe, Rutsuko Komaki, Qu Zhang

**Affiliations:** ^1^ Department of Radiotherapy Center, Xi’an International Medical Center Hospital, Xi’an, Shanxi, China; ^2^ Sapporo High Functioning Radiotherapy Center, Sapporo kojinkai Memorial Hospital, Sapporo, Japan; ^3^ Department of Radiation Oncology, Emeritus of The University of Texas M.D. Anderson Cancer Center, Baylor College of Medicine, Houston, TX, United States; ^4^ Department of Radiotherapy Center, Hubei Cancer Hospital, Tongji Medical College, Huazhong University of Science and Technology, Wuhan, China

**Keywords:** rhabdomyosarcoma, alveolar rhabdomyosarcoma, prostate, proton beam therapy, chemotherapy

## Abstract

Rhabdomyosarcoma (RMS) is a malignant tumor that is more common in children and rarely occurs in adults. Its prognosis mainly depends on the tumor stage and genetic type. In the past few decades, the survival rate of rhabdomyosarcoma has been significantly improved. Embryonal rhabdomyosarcoma (ERMS), alveolar rhabdomyosarcoma (ARMS), and pleomorphic rhabdomyosarcoma (PRMS) are common types of rhabdomyosarcoma. ERMS and ARMS are more common in children, while PRMS is more common in adults and has a poor prognosis. We report a case of a 40-year-old patient with ARMS. His chief complaint was difficulty urinating. The diagnosis was confirmed by puncture biopsy of the prostate, and pelvic lymph node metastasis was already present at the time of diagnosis. The patient underwent seven courses of chemotherapy and proton therapy and five courses of adjuvant chemotherapy. Unfortunately, 8 months after proton beam therapy, the patient showed disease progression (bone metastasis). This case illustrates the difficulties in managing late-stage prostatic alveolar rhabdomyosarcoma and is the first case reported in our hospital to be treated with proton beam therapy in an adult with ARMS of the prostate.

## Introduction

Rhabdomyosarcoma (RMS) is the most common sarcoma in children and adolescents, affecting the genitourinary tract in approximately 20% of cases (1, 2). Histologically, it has three subtypes: embryonal (ERMS), alveolar (ARMS), and pleomorphic (PRMS) (1, 2). ERMS and ARMS are more common in pediatric patients, whereas PRMS is more prevalent in adults and less common in children. ARMS, in particular, is rare in adults and has a worse prognosis compared to pediatric cases (3). Although soft tissue sarcomas account for 1% of all solid tumor malignancies in adults, RMS represents only 3% of all soft tissue sarcomas in this population (4, 5). It may arise as a primary malignancy or as part of a heterogeneous malignancy, such as a non-germ cell or teratomatous tumor (6). RMS in adults has a worse prognosis than in young children. Furthermore, the histological distribution of RMS differs between children and adults, with the pleomorphic subtype and RMS not otherwise specified (RMS NOS) being more common in adults (7). The prostate is considered an unfavorable disease site (8). Prostatic RMS accounts for less than 1% of all prostate malignancies. Prostatic ARMS is particularly rare and has been reported in only a small number of adult cases (3). Considering its rarity and aggressiveness, early diagnosis and active treatment are critical for improving patient survival.

Multimodality therapy for prostatic RMS typically includes surgery, chemotherapy, and radiation therapy (RT). RT is the most important component of the therapy. Induction chemotherapy followed by concurrent chemoradiation is the standard treatment for patients with unresected disease, micro- or macroscopic residual disease after surgery, or lymph node involvement (9, 10). Well-known optimal treatment for prostate malignancy has been divided into photon therapy and proton beam therapy (PBT). Photon therapy is often avoided due to intolerance of the surrounding organs at risk. In contrast, PBT, a form of particle therapy, offers excellent dose localization to the target volume, followed by a dose falloff beyond the tumor (11, 12). Herein, we present the clinical course of a patient treated with PBT for prostatic RMS.

## Case presentation

**Figure 1:**
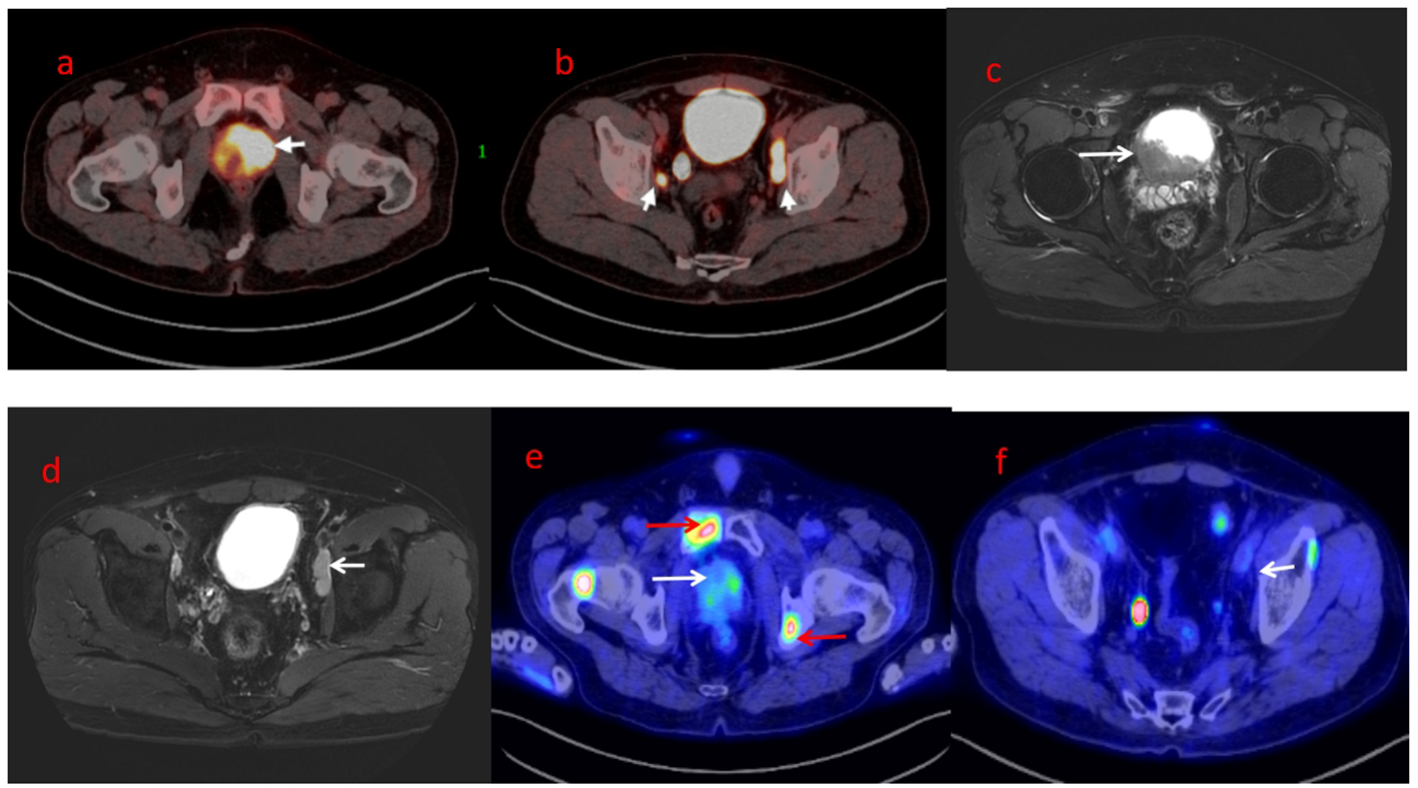
The mass infiltrates the whole prostate and the right lateral wall of the urinary bladder. The right lower ureter was infiltrated by the lesion. Large lymph nodal mass was also seen in the pelvis with multiple retroperitoneal lymphadenopathies (**a&b**, white arrow). Pelvic MRI showed suspicion of prostate cancer, invasion of the bladder base and seminal vesicles on both sides, multiple enlarged lymph nodes in the bilateral pelvis, and retroperitoneal lymph node metastasis (**c&d**, white arrow). The PET-CT 8 months after PBT showed that the prostate lesions were basically under control and the metastatic lymph nodes in the pelvis were significantly reduced (**e&f**, white arrow). PET-CT showed multiple bone metastases (**e**, red arrow).

A 40-year-old man presented with complaints of acute urinary retention for the past 6 months. He was initially diagnosed with prostatitis at a local hospital but did not receive further treatment. Later, a positron emission tomography (PET)–computed tomography (CT) scan revealed a large enhancing mass in the pelvis (**Figure 1**). The mass infiltrated the entire prostate and extended into the right lateral wall of the urinary bladder. Additionally, the lesion involved the left lower ureter. Large lymph node masses were observed in the bilateral pelvis, along with multiple retroperitoneal lymphadenopathies. The patient developed severe difficulty urinating and was subsequently given an indwelling catheter. A prostate biopsy confirmed prostatic RMS. Immunohistochemistry findings were consistent with ARMS, with Ki-67 staining showing 90% positivity. A pelvic magnetic resonance imaging (MRI) scan further supported the diagnosis, showing invasion of the bladder base and seminal vesicles on both sides, multiple enlarged lymph nodes in the bilateral pelvis, and retroperitoneal lymph node metastasis (**Figure 1**).

The treatment plan included systemic chemotherapy, consisting of VAC regimen (vincristine, actinomycin D, and cyclophosphamide) and IE regimen (ifosfamide and etoposide). The patient’s urinary catheter was removed after four cycles of chemotherapy. Following seven cycles, a slight reduction in lesion size was noted. However, repeat PET–CT still showed suspicion of prostate cancer, with invasion of the bladder base and seminal vesicles on both sides, multiple enlarged lymph nodes in the bilateral pelvis, and retroperitoneal lymph node metastasis. At this stage, the doctor recommended surgery or chemoradiotherapy. Surgery required the removal of the pelvic organs with subsequent long-term fistula maintenance, significantly impacting quality of life. Due to these concerns, the patient refused surgical treatment and instead opted for proton therapy in Japan. The patient had no history of smoking or social alcohol consumption and denied any family history of cancer.

**Figure 2:**
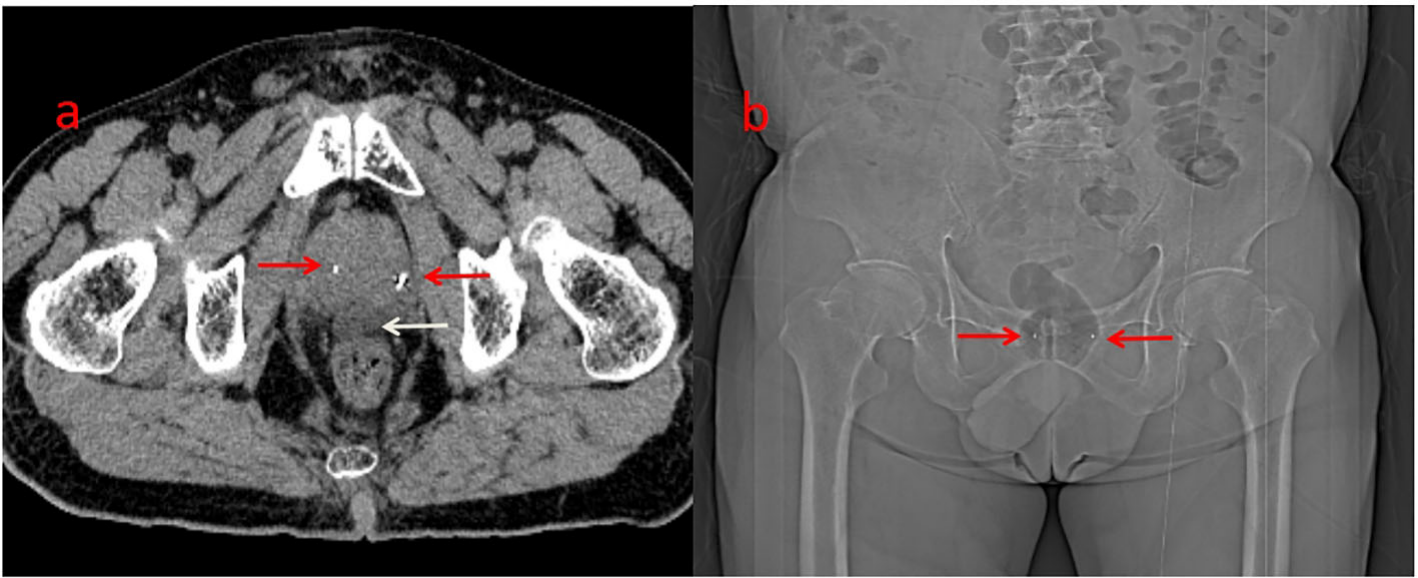
Before the protontherapy, two gold markers were inserted on the prostate (**a&b**, red arrow). Hydrogel (**a**, white arrow) was inserted between the rectum and the prostate (better to protect the rectum).

**Figure 3:**
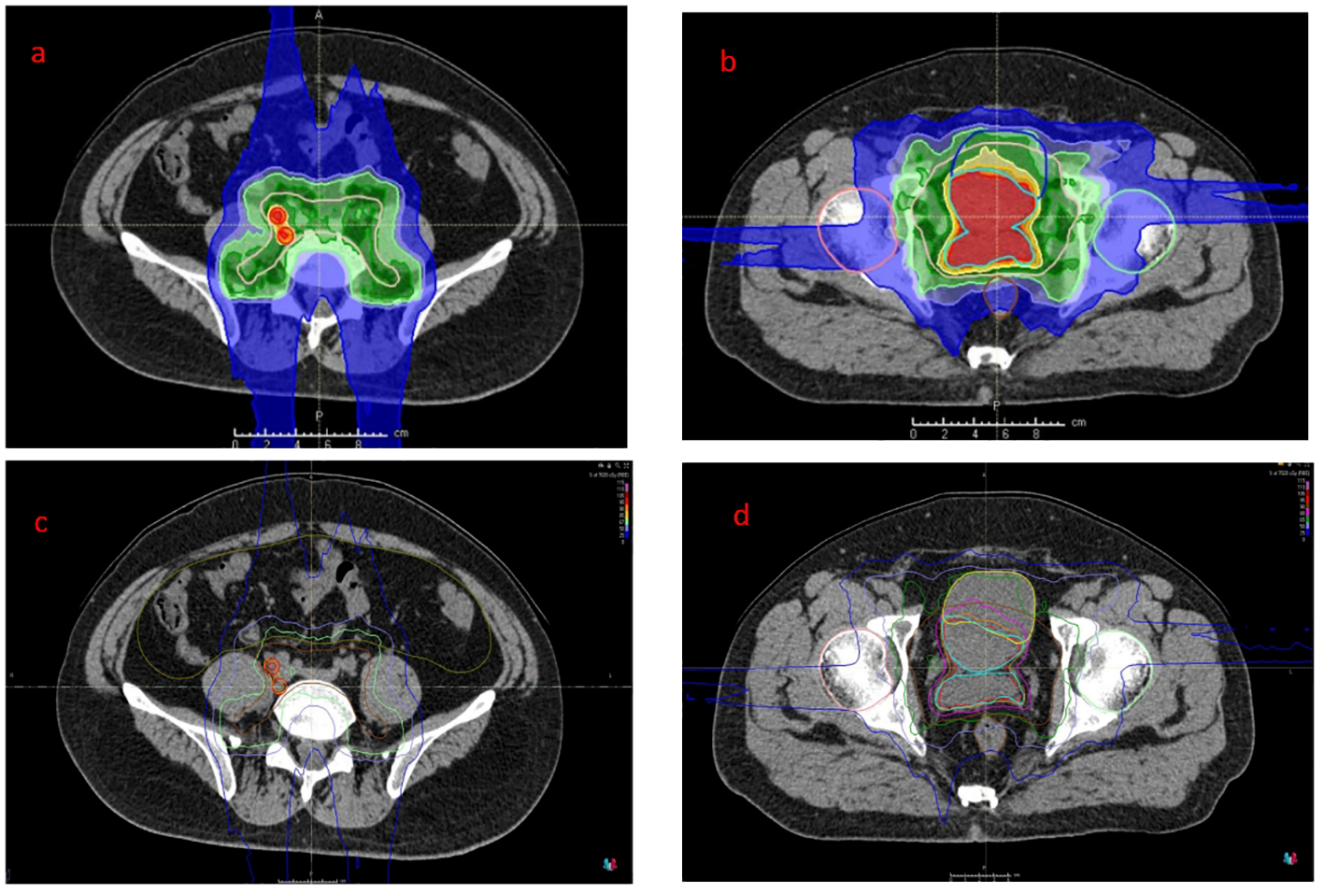
Proton dose distribution cloud map of the retroperitoneal lymphatic drainage area (**a**, dark green area), retroperitoneal metastatic lymph nodes (**a**, red area) and pelvic lymphatic drainage area (**b**, dark green area) and GTV (**b**, red area). The isodose distribution for proton therapy (**c&d**). GTV, gross target volume.

After a multidisciplinary discussion, PBT was planned for treating prostatic ARMS. Before the treatment, two gold markers were inserted into the prostate, and a Space Organ At Risk (SpaceOAR) was placed between the rectum and prostate to better protect the rectum (**Figure 2**). Gross target volume (GTV) included the prostate, bilateral seminal vesicles, and pelvic metastatic lymph nodes. Clinical target volume (CTV) included the common iliac, internal and external iliac, presacral, obturator lymphatic drainage, and primary tumors (**Figure 3**). The relative biological effectiveness (RBE) of PBT was assumed to be 1.1. We used the spot-scattering method for PBT and completed the median dose. The dose administered by GTV was 70.2 Gy (RBE) in 26 fractions (2.7 Gy per fraction), and that by CTV was 46 Gy (RBE) in 26 fractions (1.76 Gy per fraction). The dose requirement ensured that 95% of the CTV received 95% of the prescribed dose. The organs at risk (OARs) included the bladder, rectum, and small intestine. PBT was performed from May 29 to July 3, 2023. After radiotherapy, the patient returned to his home country for five cycles of chemotherapy. A PET–CT scan conducted in March 2024 showed that the prostate lesions were effectively controlled, and the metastatic lymph nodes in the pelvis had significantly reduced (**Figure 1**). Regrettably, the follow-up PET–CT 8 months after PBT revealed bone metastasis.

## Discussion

Sarcomas rarely occur in the prostate, accounting for 0.3%–1.0% of all prostate tumors, and among these, about one-third are ERMS (13). Prostatic RMS often occurs in children, predominantly the ERMS subtype. ARMS is extremely rare in adults, with only four cases reported to date (summarized in **Table 1**) (1, 3, 14, 17). ARMS generally does not cause typical symptoms but grows rapidly and spreads distantly. It can invade adjacent tissues within a short period, and the tumor is usually in an advanced stage when discovered, eliminating the possibility of surgery. The serum prostate-specific antigen (PSA) concentration mostly remains within the normal range; however, lower urinary tract obstruction symptoms, such as dysuria, frequent urination, and urgency, are common (1, 14). When the rectum is compressed, the patients develop defecation difficulties or even intestinal obstruction symptoms (15, 17). Risk factors for a poor prognosis include an age of ≥10 years, nodal involvement, tumors in unfavorable sites, and large tumors (>5 cm) (8, 17). The present case exhibited all of these risk factors, contributing to disease progression and poor treatment response.

**Table 1:** Summary of case reports of prostatic RMS in adult men.

Authors	Age/sex	Histological subtype	Molecular/cytogenetic alterations	Treatment	Clinical course	Outcome
Hans-Ulrich Schildhaus et al., 2016, *Diagnostic Pathology* (1)	25/M	ERMS	Positive for vimentin, desmin, actin, myogenin, and CD99	CT+RT	Chemotherapy, then locally aggressive plan followed by RT (but no radiotherapy)	Died 17 months after initial diagnosis
Olivas AD, et al., 2020, *Int J Surg Pathol* (3).	53/M	ERMS	Positive for desmin and negative for S100, SOX10, CDK4, MDM2, and AE1/AE3. The cytomorphology of the tumor and the lack of fusion transcripts were most consistent with ERMS	IR+CT+RT	Initial resection; postsurgical imaging 2 months later revealed pulmonary metastases; chemotherapy and pelvic radiation	Good response to chemotherapy, further course unknown
Ciammella P, et al., 2013, *Rep Pract Oncol Radiother.* (14)	27/M	ERMS	Negative for cytokeratin (AE1/AE3), CD34, S100 protein, and PSA; Ki67/MIB1 antibodies was high (>30%)	CT and IMRT	Chemotherapy and IMRT; multiple lung metastases with pleural effusion. No response was seen to palliative chemotherapy	Died from metastatic disease (pulmonary failure) 27 months following the initial diagnosis
Hanwen Luo, et al., 2024, *Journal of International Medical Research* (17)	40/M	ERMS	Positive expression: S100 protein, calcium-binding protein (caldesmon), vimentin, and desmin. Myogenin, MyoD1, anti-smooth muscle antibody, and CD34	CT	2 cycles of chemotherapy; isocyclophosphamide for injection and etoposide for injection; 2 cycles of chemotherapy	Died 4 months after initial diagnosis

ERMS, embryonal rhabdomyosarcoma; CT, chemotherapy; RT, radiotherapy; IMRT, intensity-modulated radiation therapy; IR, initial resection; PSA, prostate-specific antigen.

Pathological examination for ARMS remains the gold standard for diagnosis (3–5). Currently, the advances in molecular testing and genetic diagnostics have further improved the reliability of clinical and histological criteria for RMS classification; however, its predictive value is still limited. Molecular testing has identified key chromosomal translocations in ARMS, particularly PAX3-FKHR and PAX7-FKHR fusion transcripts (16). The majority of case reports in the literature describe cases of prostatic RMS. While only a few cases of prostatic ERMS and PRMS have been reported, several cases of prostatic ARMS in adults have been reported (17). In this case, the diagnosis of ARMS was confirmed by immunohistochemistry.

For adult patients, in great part due to the rarity of the disease and the lack of consensus on optimal treatment, the clinical outcome is still poor (18, 19). The prognosis of ARMS in children is good, while the treatment efficacy in ARMS in adults is very poor. This may be related to a variety of factors, such as the lack of standard treatment options for adult patients with ARMS. Most patients are in the late stage when they are diagnosed, with local organ infiltration or distant metastasis, and they lose the opportunity for surgical treatment, resulting in a poor prognosis. Surgical treatment remains the primary method of treatment for ARMS, with postoperative adjuvant chemotherapy and/or radiotherapy, but most adult patients with ARMS are in the late stage, making surgery a challenging option due to the influence of multiple factors such as tumor size and poor location (18). The clinical approach is particularly complex at specific tumor sites. A good example is RMS arising in the bladder or rectum or the associated urinary cavity has lymphadenopathy and metastasis; in such cases, achieving negative margins becomes challenging. Resection in these areas may cause important functional damage to vital surrounding organs and can severely compromise cosmesis. Therefore, chemotherapy and radiation therapy are the main methods of treatment; however, the effectiveness may not be uniform. Induction chemotherapy, followed by concurrent chemoradiotherapy, is the current standard of treatment for patients with unresected disease, micro- or macroscopic residual disease after surgery, or lymph node involvement and those with alveolar histology (2, 10). However, chemoradiotherapy is usually not very effective.

Of the treatment options for prostate malignancy, PBT offers a distinct physical advantage due to the Bragg peak phenomenon: it delivers minimal energy when passing through normal tissues and releases a high dose at the tumor site, with a sharp dose falloff beyond the tumor (20). The RBE of proton therapy is usually 1.1, which means that it has 10% greater effectiveness than photon therapy. PBT can increase the radiation dose to the tumor while minimizing the dose to normal tissues, thereby potentially improving local control, reducing toxicity, and improving quality of life (18–21). In this case, the patient underwent PBT for prostatic ARMS. The dose for GTV and CTV for the clinical target was 70.2 Gy (RBE) and 46 Gy (RBE), respectively. The patient completed all treatments within 4 weeks and had no other discomfort, except for occasional frequent urination. The patient’s rectal dose was significantly reduced, likely due to SpaceOAR placement before radiotherapy. Because the bladder was invaded by the tumor, part of the bladder was included in the target volume, resulting in a slightly higher bladder dose. The patient returned home with the prostate lesions under control, which required more aggressive systematic treatment. This case highlights both the efficacy and limitations of PBT in treating prostatic ARMS. The findings underscore the highly aggressive nature of ARMS.

RMS is a heterogeneous disease in both clinical presentation and histological characteristics. With the ongoing development and optimization of multimodal treatment strategies, therapeutic approaches for prostatic RMS have incorporated neoadjuvant/adjuvant chemotherapy, radiotherapy, and radical surgery, notably improving patient survival rates and reducing mortality. However, survival rates remain poor for patients diagnosed with widely metastatic or recurrent RMS (22, 23). Most patients present with large tumors at diagnosis, frequently accompanied by metastases or lesions that are challenging to completely resect (24). Although advances in multimodal treatment have improved overall survival rates for low- and intermediate-risk patients, treatment efficacy for metastatic disease remains limited.

In the era of modern targeted immunotherapy, the focus of RMS treatment has gradually shifted from radical surgery to a combination of chemotherapy, targeted therapy, immunotherapy, and radiotherapy to achieve effective tumor control owing to the difficulty of achieving complete resection or the high likelihood of recurrence. RMS remains a rare malignancy, and research into combination targeted therapies is largely confined to preclinical stages, with limited clinical investigation, most of which focuses on pediatric RMS. For example, the Children’s Oncology Group conducted a Phase II trial (ARST0431) to assess the feasibility of an “interval compression” strategy, which demonstrated improved progression-free survival in some children with metastatic RMS (25). Additionally, in the Phase II pilot study, ARST0921, the incorporation of the anti-angiogenic agent bevacizumab or the mTOR inhibitor temsirolimus resulted in a slight improvement in efficacy with temsirolimus (26).

Radiotherapy not only exerts direct cytotoxic effects on cancer cells but also promotes tumor antigen recognition at distant sites, activating T cell-mediated suppression of untreated tumors (27). The combination of radiotherapy and immunotherapy has demonstrated significant synergistic effects, enhancing both local and systemic tumor control. Recent advances indicate that this combination offers promising therapeutic outcomes (28). Although the combination of proton therapy and immunotherapy remains under investigation, preliminary studies have reported favorable synergistic effects. In specific cases, the concurrent administration of immune checkpoint inhibitors during proton beam therapy or sequential use of immunotherapy alongside targeted therapy and chemotherapy may contribute to the suppression of distant metastasis.

In conclusion, targeted therapies and immunotherapies represent promising treatment options for RMS. When combined with chemotherapy or radiotherapy, these approaches may offer therapeutic benefits by reducing the risk of distant metastasis and recurrence. However, their efficacy requires further validation through large-scale clinical studies.

## Conclusions

Prostatic ARMS is an exceptionally rare malignancy with limited treatment options and a poor prognosis in adults. Therefore, through this case report, we aim to elucidate several issues. First, ARMS should be considered in the differential diagnosis so that it can be diagnosed promptly at an early stage. Second, multidisciplinary treatment can improve the outcome of patients. Third, prostatic ARMS treated with PBT shows good local control but can quickly develop distant metastases, indicating the need to strengthen more aggressive systemic treatment. Finally, the outcome of prostatic ARMS is poor because of the lack of appropriate diagnostic and treatment guidelines. For complex and refractory prostatic RMS, future clinical trials should focus on combining radiotherapy with novel immunotherapeutic strategies to improve outcomes for adult patients with ARMS.

## Data Availability

The original contributions presented in the study are included in the article/supplementary material. Further inquiries can be directed to WY, ywencui@163.com.
